# LncRNA GAS6-AS1 facilitates tumorigenesis and metastasis of colorectal cancer by regulating TRIM14 through miR-370-3p/miR-1296-5p and FUS

**DOI:** 10.1186/s12967-022-03550-0

**Published:** 2022-08-12

**Authors:** Qing Chen, Lin Zhou, De Ma, Juan Hou, Yuxin Lin, Jie Wu, Min Tao

**Affiliations:** 1 Department of Oncology, Jingjiang People’s Hospital, The Seventh Affiliated Hospital of Yangzhou University, Jingjiang, Jiangsu China; 2 Department of Oncology, The First Affiliated Hospital of Soochow University, Suzhou, Jiangsu China; 3 Department of Urology, The First Affiliated Hospital of Soochow University, Suzhou, Jiangsu China; 4 Department of Oncology, Dushu Lake Hospital Affiliated to Soochow University, Suzhou, Jiangsu China

**Keywords:** Long non-coding RNAs, GAS6-AS1, TRIM14, FUS, miR-370-3p, miR-1296-5p

## Abstract

**Background:**

Long non-coding RNAs (lncRNAs) are essential regulators of tumorigenesis and the development of colorectal cancer (CRC). Here, we aimed to investigate the role of lncRNA GAS6-AS1 in CRC and its potential mechanisms.

**Methods:**

Bioinformatics analyses evaluated the level of GAS6-AS1 in colon cancer, its correlation with clinicopathological factors, survival curve and diagnostic value. qRT-PCR were performed to detect the GAS6-AS1 level in CRC samples and cell lines. The CCK8, EdU, scratch healing, transwell assays and animal experiments were conducted to investigate the function of GAS6-AS1 in CRC. RNA immunoprecipitation (RIP) and dual-luciferase reporter gene analyses were carried out to reveal interaction between GAS6-AS1, TRIM14, FUS, and miR-370-3p/miR-1296-5p.

**Results:**

GAS6-AS1 was greatly elevated in CRC and positively associated with unfavorable prognosis of CRC patients. Functionally, GAS6-AS1 positively regulates CRC proliferation, migration, invasion, and epithelial-mesenchymal transition (EMT) in vitro and induces CRC growth and metastasis in vivo. Moreover, GAS6-AS1 exerted oncogenic function by competitively binding to miR-370-3p and miR-1296-5p, thereby upregulating TRIM14. Furthermore, we verified that GAS6-AS1 and TRIM14 both interact with FUS and that GAS6-AS1 stabilized TRIM14 mRNA by recruiting FUS. Besides, rescue experiments furtherly demonstrated that GAS6-AS1 facilitate progression of CRC by regulating TRIM14.

**Conclusion:**

Collectively, these findings demonstrate that GAS6-AS1 promotes TRIM14-mediated cell proliferation, migration, invasion, and EMT of CRC via ceRNA network and FUS-dependent manner, suggesting that GAS6-AS1 could be utilized as a novel biomarker and therapeutic target for CRC.

**Supplementary Information:**

The online version contains supplementary material available at 10.1186/s12967-022-03550-0.

## Background

Colorectal cancer (CRC) is among the most prevalent malignant tumors and accounts for nearly 10% of annual cancer diagnoses globally. Additionally, CRC is the main identifiable cause of cancer-related deaths globally [1, 2]. Recent advancements in surgery, radiotherapy, chemotherapy, targeted therapy, immunotherapy, and multimodal therapy have led to significant progress in CRC treatment. However, the overall survival of patients with advanced CRC remains poor. Tumor-associated recurrence and metastasis are the main causes of death in CRC patients [3]. Consequently, there is an urgent need to identify practical and reliable biomarkers and explore the underlying disease mechanisms to aid in CRC diagnosis and therapy.

Long non-coding RNAs (lncRNAs), which are RNA transcription products comprised of more than 200 nucleotides, cannot encode proteins. LncRNAs regulate gene expression and function at the transcriptional, translational, and post-translational levels [4]. Compelling evidence shows that lncRNAs are largely associated with the occurrence and development of numerous diseases including tumors, metabolic disorders, and cardiovascular diseases [5]. Recent studies demonstrate the role of lncRNAs in tumor pathogenesis, including cell proliferation, migration, invasion, epithelial-mesenchymal transition (EMT), apoptosis, drug resistance, and immune escape [6, 7]. Notably, lncRNA GAS6-AS1 is the antisense transcriptional RNA of growth arrest specific 6 (GAS6). GAS6-AS1 regulated GAS6 level during transcription or translation phase, thereby boosting the AXL receptor tyrosine kinase (AXL) level and stimulating AXL signal. GAS6 and GAS6-AS1 are both involved in the pathogenesis of cancers [8]. Although most studies revealed that GAS6-AS1 functions as an oncogene and was highly expressed in gastric, hepatocellular, breast cancers and acute myeloid leukemia (AML), there were studies found that low GAS6-AS1 expression was associated with poor prognosis in lung cancer patients and that GAS6-AS1 overexpression inhibited lung adenocarcinoma progression [9–15]. However, the role of GAS6-AS1 in CRC remains unclear.

RNA binding proteins (RBPs) play key roles in post transcriptional level by specifically binding to RNAs, affecting the cellular process of cancers [16]. Fusion in sarcoma/liposarcoma (FUS), as a tumorigenesis-related RBP, which involved in transcription regulation and RNA processing, has been previously reported in multiple cancers, including CRC [17–20].

Here, we reveal that GAS6-AS1 is upregulated in CRC and that elevated GAS6-AS1 expression is associated with unfavorable prognosis in CRC patients. Functional experiments demonstrated that GAS6-AS1 exerts an oncogenic role by promoting CRC growth and metastasis. Mechanistically, GAS6-AS1 promotes CRC tumorigenesis by acting as a competitive endogenous RNA (ceRNA) for miR-370-3p and miR-1296-5p which contributes to TRIM14 upregulation. Furthermore, GAS6-AS1 stabilizes TRIM14 mRNA in an FUS-dependent manner. Thus, we show the clinical significance of GAS6-AS1 in CRC and its underlying mechanism, thereby providing new insights into CRC tumorigenesis.

## Materials and methods

### Database analysis

GAS6-AS1 expression data and related clinical information were extracted from the TCGA-COAD dataset using TCGA database (https://cancergenome.nih.gov/). These data were analyzed using R software (3.5.1). The GAS6-AS1-targeted miRNAs and the binding site were predicted using StarBase (http://starbase.sysu.edu.cn/index.php). The binding site between miR-370-3p/miR-1296-5p and TRIM14 was predicted using TargetScan (http://www.targetscan.org/vert_72/).

### Clinical samples

Paired CRC and para-carcinoma samples were obtained from 40 patients who underwent tumor resection at the First Affiliated Hospital of Soochow University (November 2019 to May 2020). Each patient was CRC-positive at diagnosis and had not received preoperative chemoradiotherapy. The age of patients was range from 40 to 75-year-old. Informed consent was obtained from all patients. This study was approved by the ethics committee of the First Affiliated Hospital of Soochow University (No.2019138).

### Cell culture

The human CRC cell lines HT29, LoVo, RKO, SW620, the human normal colon epithelial cells (NCM460), and the human embryo kidney cell line HEK-293T were purchased from the Cell Bank of the Chinese Academy of Sciences and the American Type Culture Collection (ATCC). The cells were cultured in RPMI-1640 (Hyclone, USA) or DMEM medium (Hyclone, USA) with 10% fetal bovine serum in a cell incubator at 37 °C and 5% CO_2_.

### Real-time RT-PCR

Total RNA was extracted using a TRIzol reagent kit (Invitrogen, USA). Primers were designed by Sangon Biotech (Shanghai, China) and were listed in Additional file 1: Table S1. Quantitative real-time PCR was conducted with either 2X SYBR Green qPCR Master Mix (Abm, Canada) or miDETECT A Track miRNA qRT-PCR Starter Kit (RiboBio, China). β-actin (for mRNAs and lncRNAs) and U6 (for miRNAs) served as controls.

### Subcellular fractionation

The PARIS Kit (Invitrogen, USA) was utilized to extract the cell nuclear and cytoplasmic RNA, for subsequent qRT-PCR. We used β-actin (for cytoplasm) and U6 (for nuclear) for normalizations.

### Generation of cell lines with stable overexpression and knockdown of GAS6-AS1

The pCDH-GAS6-AS1, pCDH (blank plasmid), pLenti hU6/shRNA-GAS6-AS1, and pLenti hU6/shRNA-Control was established by Lingke Biotechnology (Shanghai, China). Then, plasmids were transfected into with HEK-293T cells psPAX2 and pMD2G. Virus particles were sterile-filtered, concentrated, and subsequently used for infecting HT29 and LoVo cells to produce corresponding stable cells.

### Cell transfection

The miR-370-3p mimics, miR-1296-5p mimics, miR-370-3p inhibitors, miR-1296-5p inhibitors and matched negative controls were synthetized by RiboBio (Guangzhou, China). Small interfering TRIM14 (si-TRIM14, CCA CAT GTG GGT ACT GCA T) were purchased from RiboBio. Lipofectamine 2000 (Invitrogen, USA) was applied for transfection following manufacturer's guide.

### Cell proliferation assay

Cells were grown in 96-well plate with 5 × 10^3^ per well. Cell proliferation was detected by Cell Counting Kit (Beyotime, China) after transfected or not. Then the absorbance at 450 nm was measured.

### EdU assays

BeyoClick EdU-555 Kits (Beyotime, China) were used for EdU assays. Cells were cultivated in medium containing 10 μM EdU before fixing with 4% paraformaldehyde and subsequent stained with EdU reaction buffer. To visualize the DNA, the cells were stained with Hoechst and observed with fluorescence microscope. The EdU-positive cells were counted.

### Scratch healing assay

Cells were seeded into 24-well plates and cultured in the incubator. 10 μl pipette tips were used to scratch on monolayer cells at multiple sites. The area of scratch was observed at 0 h, 24 h and 48 h after scratching. Resulting images were processed with ImageJ software.

### Transwell assays

Transwell chambers (Corning, USA) with (migration) and without (invasion) Matrigel (BD Biosciences, USA) were applied to perform transwell assays. Briefly, 1 × 10^5^ cells were cultured in the upper wells with 100 µL serum-free medium. The lower chamber was infused with 600 µL complete medium. After 48 h, the cells on the lower side of the membrane were fixed in 4% paraformaldehyde, stained with 0.1% crystal violet, and quantified under a microscope.

### Experimental animals

Animal experiments were conducted following the principles of the Animal Management and Use Committee of Soochow University and approved by the Medical Ethics Committee of The First Affiliated Hospital of Soochow University (Approval No.2017213). 24 BALB/c nude mice (4–5 weeks) were obtained from SLAC Laboratory Animal Center (Shanghai, China) and reared in a specific-pathogen-free environment at 23–25 °C. Xenograft tumors were established by subcutaneously injecting 5 × 10^6^ cells (HT29-GAS6-AS1, HT29-Vector, LoVo-shGAS6-AS1, and LoVo-shControl cells). The tumor size was determined weekly. The mice were euthanized after four weeks, and the tumors were excised and weighed.

For lung metastasis models, 2 × 10^6^ cells (HT29-GAS6-AS1 vs. HT29-Vector, LoVo-shGAS6-AS1 vs. LoVo-shControl) were injected through the tail vein. The mice were euthanized four weeks later. The lung tissue was dissected and fixed with formalin. Lung foci were counted using H&E staining.

### RNA fluorescent in situ hybridization

Fluorescent in situ hybridization (FISH) kit (RiboBio, China) was applied for the in-situ detection of GAS6-AS1 in HT29 and LoVo cells following the guidelines. Cells were observed by the fluorescence microscope.

### Dual-luciferase reporter assay

For dual-luciferase reporter assays, the wild-type (WT) 3′-UTR of GAS6-AS1/TRIM14 gene containing miR-370-3p and miR-1296-5p binding sites, and the mutant (MUT) 3′-UTR of GAS6-AS1/TRIM14 gene were obtained from RiboBio (Guangzhou, China). The WT/MUT 3′-UTR were co-transfected with mimics or negative control with Lipofectamine 2000 (Invitrogen, USA). Notably, 48 h post-transfection, cells were lysed. Then, we applied the Dual-Luciferase Reporter Assay Kit (Beyotime, China) to evaluate the luciferase activities.

### RNA immunoprecipitation (RIP)

RIP was performed using EZ-Magna RIP kits (Millipore, USA). Cells were collected and incubated with anti-Ago2 antibody (Abcam, UK) 24 h after transfection. IgG was used as the negative control. Quantitative RT-PCR was used to assess the co-precipitated RNAs.

### Western blot analysis

Here, cells were solubilized in RIPA lysis buffer (Merck, China.). With the BCA method, we standardized the protein concentration. Lysates were resolved by 10–15% SDS polyacrylamide gels and transferred onto PVDF membranes. The membranes were incubated overnight at 4 °C with primary antibodies, including anti-TRIM14 (1:500, Proteintech, USA), anti-GAPDH (1:5000, CST, USA), and anti-E-cadherin (1:1000), anti-N-cadherin (1:1000), anti-Vimentin (1:1000) (Immunoway, USA), followed by another incubation with appropriate secondary antibodies (the anti-rabbit or anti-mouse antibodies were from Immunoway) at 25 °C 2 h. The blots were developed using ECL.

### Statistical analysis

All data were analyzed using Prism 7.0 or SPSS24.0. T-test or ANOVA analyses were applied to examine the differences among the groups. *P* < 0.05 denoted a statistical difference.

## Results

### GAS6-AS1 is overexpressed in CRC and correlates with poor clinical outcome

**Fig. 1:**
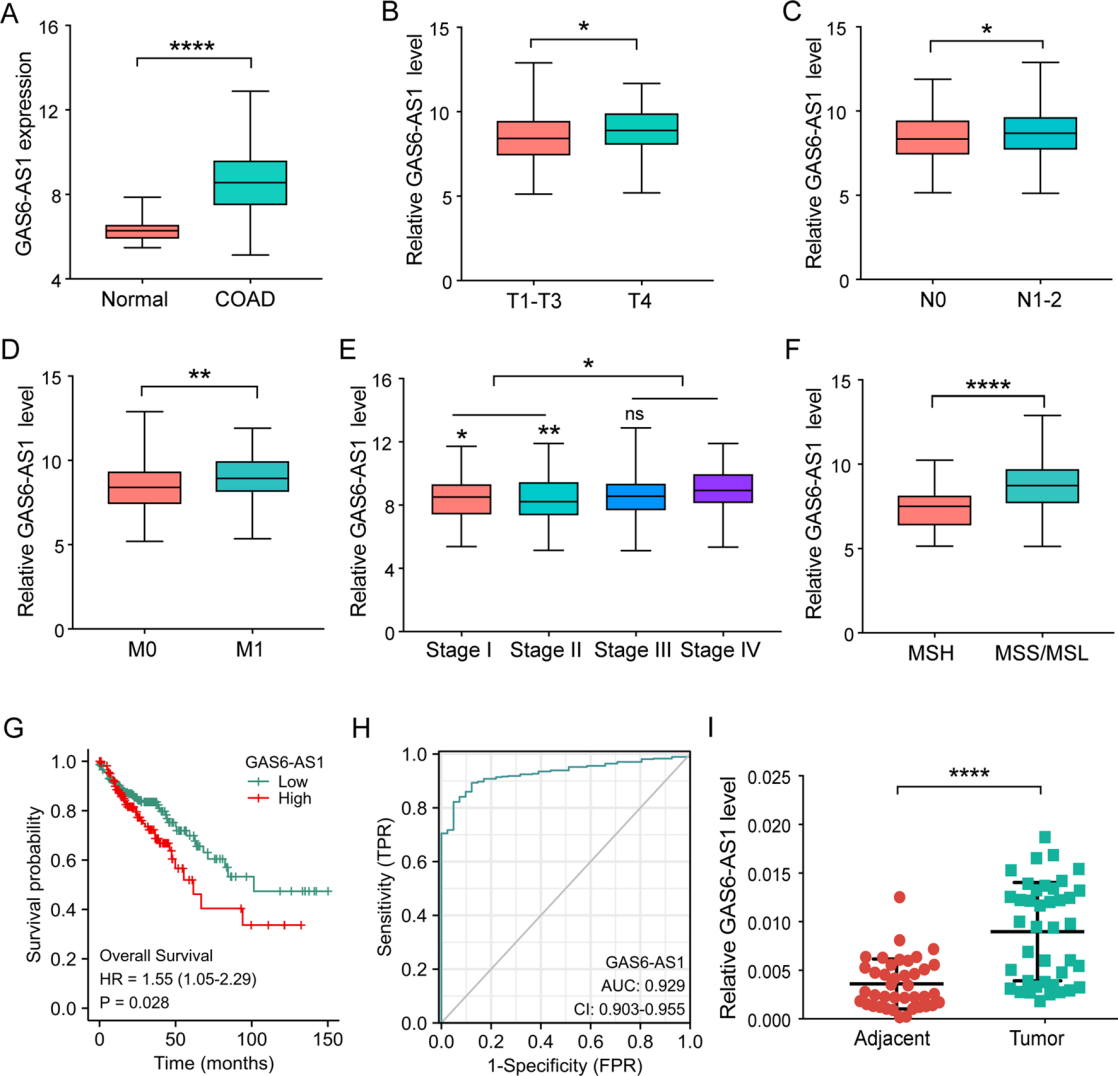
GAS6-AS1 was highly expressed in CRC and associated with poor prognosis. **A** GAS6-AS1 expression in COAD compared with normal tissues from the TCGA database. **B** GAS6-AS1 expression in T1-3 stage patients compared with T4 stage. **C** GAS6-AS1 expression in N0 stage patients compared with N1-2 stage. **D** GAS6-AS1 expression in M0 and M1 stage patients. **E** GAS6-AS1 expression in I-IV stage patients. **F** GAS6-AS1 expression in patients with MSI and patients with MSS/MSL. **G** Kaplan–Meier analysis of overall survival in patients grouped by expression level of GAS6-AS1 (High vs. Low). **H** The ROC curve and the AUC distribution of GAS6-AS1.** I** GAS6-AS1 expression in paired CRC tissues was examined by qRT-PCR. * *P* < 0.05, ** *P* < 0.01, **** *P* < 0.0001

Data on lncRNA GAS6-AS1 expression and corresponding clinical information were obtained from the TCGA-COAD dataset in the TCGA database, including 41 normal colon tissues and 446 colon cancer tissues. Detailed information on colon cancer samples is shown in Additional file 2: Table S2, including sex, age, T stage, N stage, M stage, clinical stage, and microsatellite instability (MSI) status (MSH: high microsatellite instability; MSS: microsatellite instability low). Expression differences and clinical prognostic analyses were performed using R software. The GAS6-AS1 level in colon cancer tissues was significantly higher than that in normal tissues (Fig. 1A). In addition, the relative GAS6-AS1 level of patients with T4 stage disease was higher than that of patients with T1-3 stage disease (Fig. 1B). GAS6-AS1 levels were higher in lymphatic metastasis-positive patients than those in lymphatic metastasis-negative patients (Fig. 1C). Furthermore, GAS6-AS1 levels in patients with distant metastasis were significantly higher than those in patients without distant metastasis (Fig. 1D). Although no statistical significance was detected between GAS6-AS1 in stage III and stage IV patients, stage IV patients exhibited higher GAS6-AS1 levels than stage I and II patients. GAS6-AS1 expression in patients with stage III-IV was higher than that in stage I and II patients (Fig. 1E). Patients with MSS and MSL exhibited higher GAS6-AS1 expression levels than patients with MSH (Fig. 1F). Survival analysis revealed that patients with relatively high GAS6-AS1 expression had shorter survival times than those with relatively low GAS6-AS1 expression (*P* = 0.028) (Fig. 1G). Receiver operating characteristic (ROC) analysis of GAS6-AS1 was performed and the area under the curve (AUC) was 0.929 (Fig. 1H), which indicates the diagnostic value of GAS6-AS1. After examining GAS6-AS1 levels in CRC, 40 pairs of operable CRC and para-cancer matched samples were collected and analyzed via qRT-PCR. The results indicated remarkably higher GAS6-AS1 levels in the CRC samples than in the para-cancerous samples (Fig. 1I). These findings suggested that GAS6-AS1 expression was elevated in CRC and that GAS6-AS1 was positively associated with tumor progression and poor prognosis.

### GAS6-AS1 promotes CRC cell viability and mobility

**Fig. 2:**
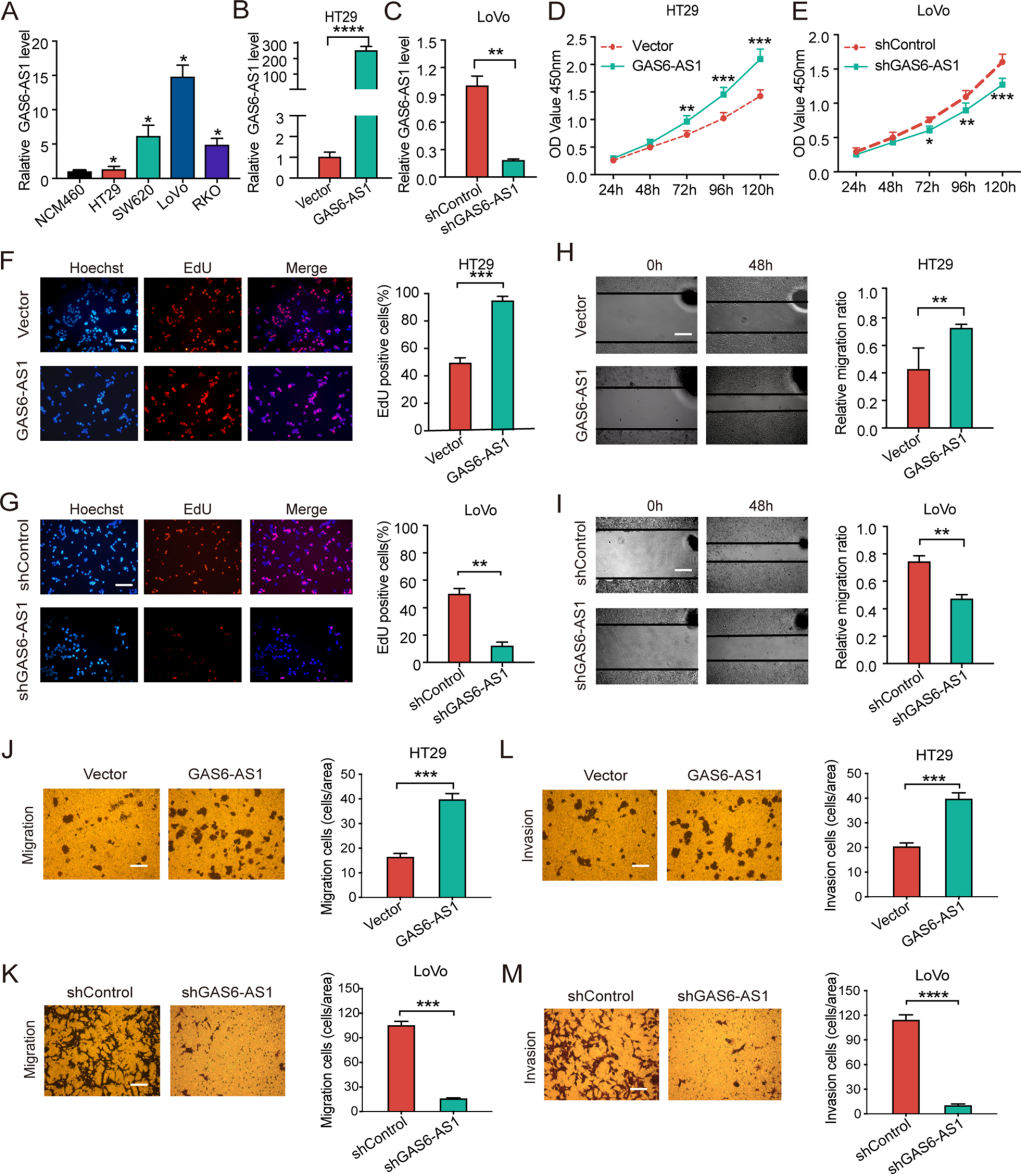
GAS6-AS1 promotes the proliferation, migration and invasion of CRC cells. **A** The expression of GAS6-AS1 of the CRC cell lines were detected via qRT-PCR. **B** After upregulation of GAS6-AS1 in HT29 cell lines, the efficiency was verified via qRT-PCR. **C** After downregulation of GAS6-AS1 in LoVo cell lines, interference efficiency was verified via qRT-PCR. **D** After upregulation of GAS6-AS1, CCK8 assay revealed that the proliferation of HT29 cells was significantly enhanced. **E** After downregulation of GAS6-AS1, CCK8 assay revealed that the proliferation of LoVo cells was significantly decreased. **F** After upregulation of GAS6-AS1, EdU assay revealed that the proliferation of HT29 cells was significantly enhanced. **G** After downregulation of GAS6-AS1, EdU assay revealed that the proliferation of LoVo cells was significantly decreased. **H**–**M** Overexpression of GAS6-AS1 significantly enhanced the migration **H**, **J** and invasion **L** ability of HT29 cells. Knockdown of GAS6-AS1 significantly suppressed the migration **I**, **K** and invasion **M** ability of LoVo cells. (scale bar: 200 μm for EdU assay, 50 μm for wound healing assay, 100 μm for Transwell assay). * *P* < 0.05, ** *P* < 0.01, *** *P* < 0.001, **** *P* < 0.0001

GAS6-AS1 levels in human CRC cell lines (HT29, SW620, LoVo, and RKO cells) and NCM460 cells were evaluated using qRT-PCR. Elevated GAS6-AS1 expression was observed in CRC cells (Fig. 2A). LoVo cells, which had the highest GAS6-AS1 levels, and HT29 cells, which had the lowest GAS6-AS1 levels, were selected for in vitro experiments. To investigate whether GAS6-AS1 influences CRC cell viability and mobility, we overexpressed GAS6-AS1 in HT29 cells (Fig. 2B) and inhibited GAS6-AS1 expression in LoVo cells (Fig. 2C). CCK8 assays demonstrated that the viability of GAS6-AS1-overexpressing HT29 cells was significantly increased (Fig. 2D), whereas GAS6-AS1 knockdown significantly suppressed LoVo cell proliferation (Fig. 2E). EdU experiments demonstrated that HT29 cell viability was greatly increased after GAS6-AS1 overexpression (Fig. 2F), whereas LoVo cell viability greatly decreased after GAS6-AS1 knockdown (Fig. 2G).

To further uncover the function of GAS6-AS1 in CRC cell mobility, scratch and transwell assays were performed. In scratch assays, we found that GAS6-AS1 upregulation significantly increased the migration of HT29 cell (Fig. 2H), whereas GAS6-AS1 downregulation reduced the migration of LoVo cell (Fig. 2I). In line with these results, transwell migration experiments revealed that the number of migrating HT29 cells increased after GAS6-AS1 overexpression (Fig. 2J), while the number of migrating LoVo cells was reduced after GAS6-AS1 knockdown (Fig. 2K). Furthermore, transwell invasion assays demonstrated that GAS6-AS1 overexpression promoted the invasion of HT29 cells (Fig. 2L), whereas downregulating GAS6-AS1 impeded LoVo cell invasion (Fig. 2M). These results indicated that GAS6-AS1 promoted CRC cell viability and mobility in vitro.

### GAS6-AS1 promotes in vivo tumorigenesis and CRC metastasis

**Fig. 3:**
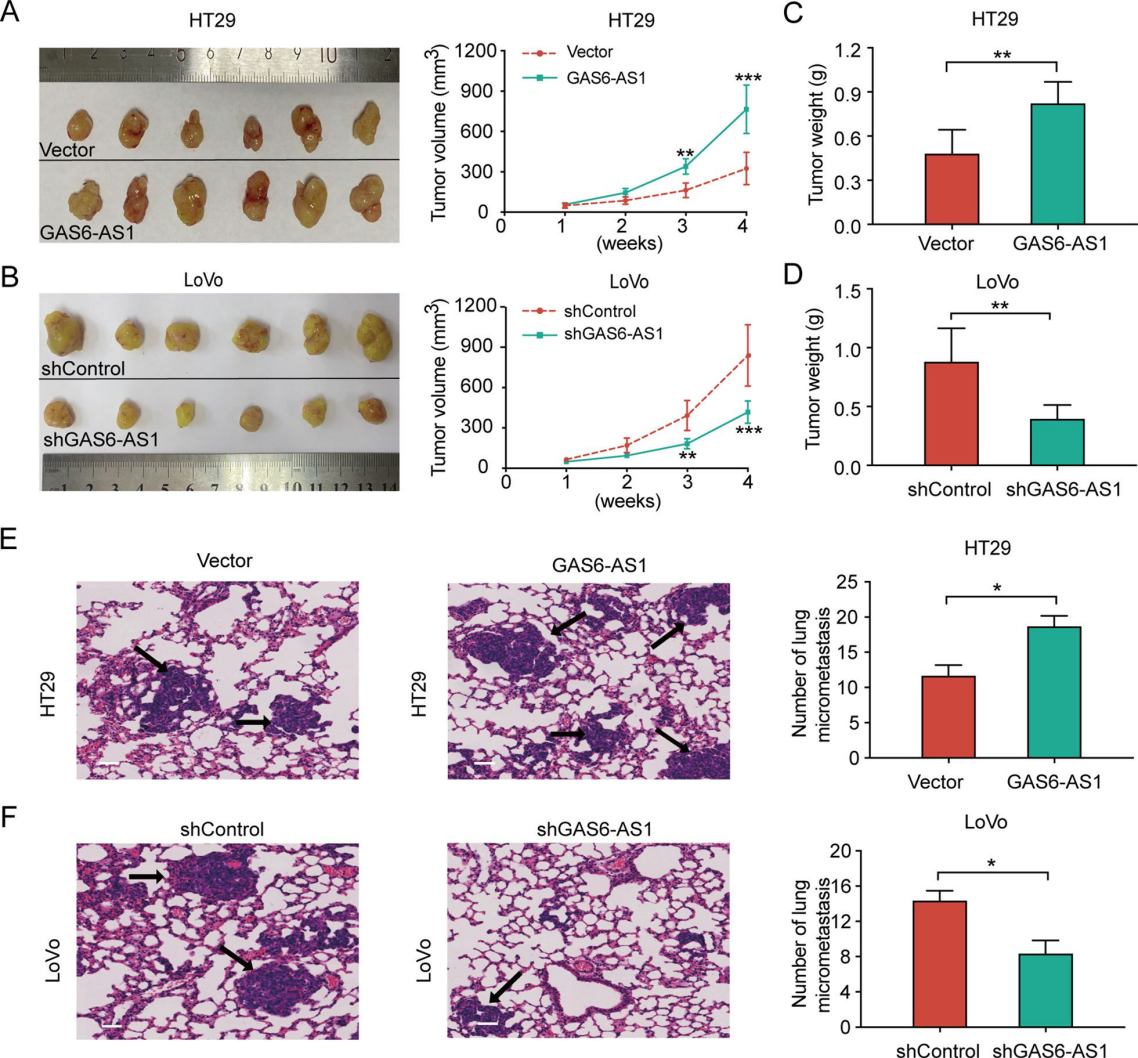
GAS6-AS1 promotes CRC tumorigenesis and metastasis in vivo.** A** Representative images and the volume of tumors derived from HT29 cells with or without GAS6-AS1 overexpression. **B** Representative images and the volume of tumors derived from LoVo cells with or without GAS6-AS1 inhibition. **C**, **D** The weight of formed tumors. **E** HT29 cells with or without GAS6-AS1 overexpression were injected into the tail vein of mice to construct lung metastasis models, the representative images and numbers of metastatic nodules in the lungs. **F** LoVo cells with or without GAS6-AS1 knockdown were injected into the tail vein of mice to construct lung metastasis models, the representative images and numbers of metastatic nodules in the lungs. The tumor micro metastasis nodules in the lungs were counted according to H&E staining. (scale bar: 50 μm). ** *P* < 0.01, ** *P* < 0.01, *** *P* < 0.001

To further evaluate the role of GAS6-AS1 in CRC tumorigenicity in vivo, xenograft models were established. Notably, GAS6-AS1 overexpression promoted subcutaneous xenograft tumor growth (Fig. 3A), whereas GAS6-AS1 knockdown significantly reduced tumor volume (Fig. 3B). Tumors of the GAS6-AS1 overexpression group weighed more than control tumors (Fig. 3C). Lower tumor weight was observed in the GAS6-AS1 knockdown group compared to the control group (Fig. 3D). To further reveal the effect of GAS6-AS1 on CRC metastasis in vivo, we developed lung metastasis models of CRC and lung metastatic foci were counted. Compared to the control group, GAS6-AS1 overexpression resulted in more metastatic pulmonary nodules (Fig. 3E). GAS6-AS1 knockdown resulted in fewer metastatic pulmonary nodules (Fig. 3F). Collectively, these observations demonstrated that GAS6-AS1 overexpression facilitated CRC growth and metastasis in vivo.

### GAS6-AS1 serves as a ceRNA and sponges miR-370-3p/miR-1296-5p

**Fig. 4:**
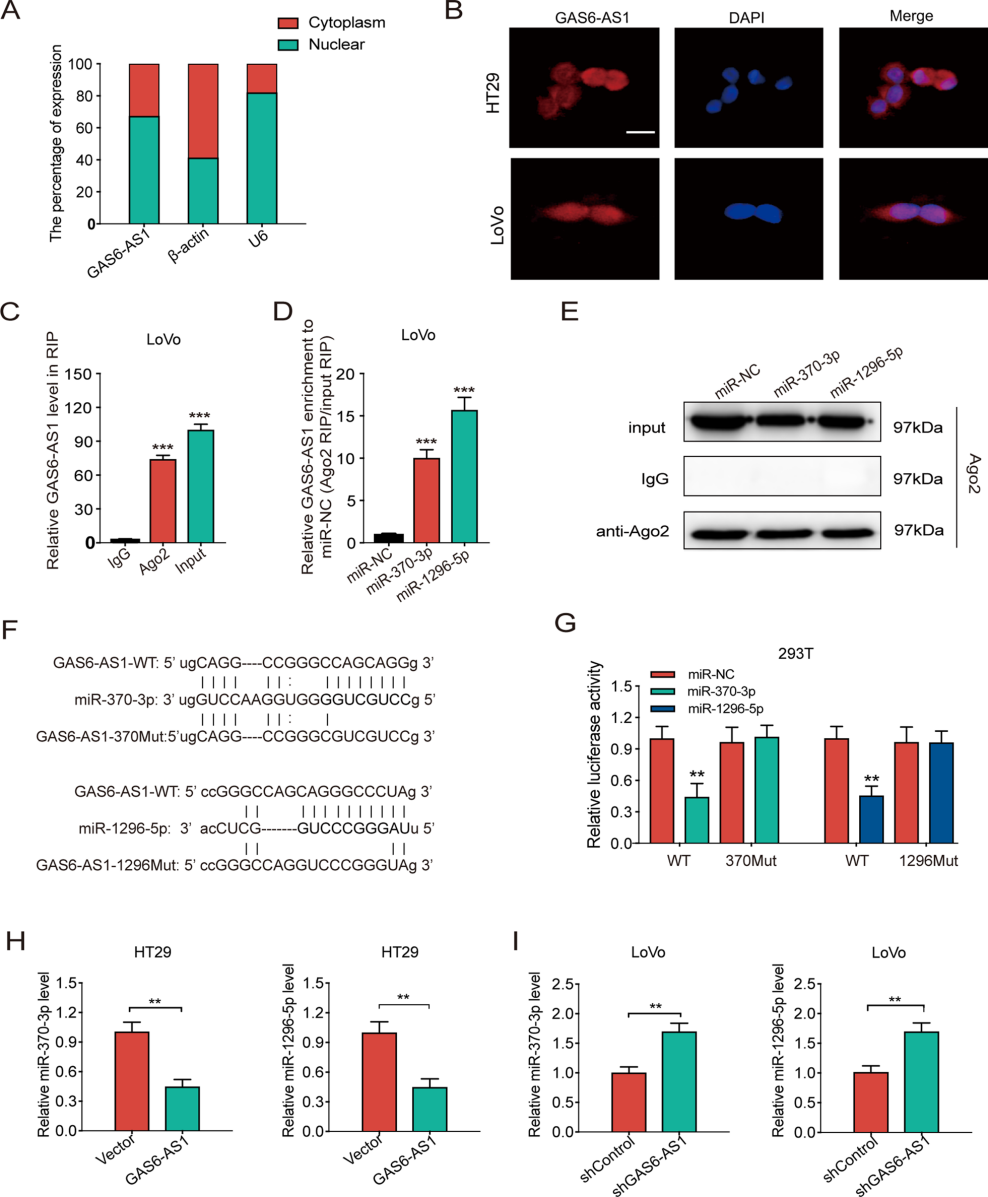
GAS6-AS1 serves as a ceRNA and sponges miR-370-3p and miR-1296-5p. **A** Subcellular localization of GAS6-AS1 was detected by qRT-PCR in LoVo cells. **B** Subcellular localization of GAS6-AS1 in HT29 and LoVo cells determined by RNA-FISH (scale bar: 20 μm). **C** Fold enrichment of GAS6-AS1 in LoVo. **D** Enrichment of GAS6-AS1 in LoVo cells transfected with miR-370-3p mimic, miR-1296-5p mimic or miR-NC. **E** Ago2 protein immunoprecipitated by Ago2 antibody or IgG was meatured by western blot. **F** miR-370-3p/miR-1296-5p and GAS6-AS1 binding sequences and GAS6-AS1mutation sequences. **G** The luciferase activities in 293T cells co-transfected with wild-type (WT) or mutant (370Mut/1290Mut) GAS6-AS1 plasmid together with miR-370-3p or miR-1296-5p mimic or miR-NC. **H** qRT-PCR showed that miR-370-3p and miR-1296-5p level were decreased when GAS6-AS1 overexpressed. **I** qRT-PCR showed that miR-370-3p and miR-1296-5p level were upregulated when GAS6-AS1 knockdown. ** *P* < 0.01, *** *P* < 0.001

To uncover cellular GAS6-AS1 functions, quantitative RT-PCR and fluorescent in situ hybridization (FISH) were performed to predict its subcellular localization. qRT-PCR demonstrated that GAS6-AS1 was localized both in the cytoplasm and nuclei of CRC cells. This observation was verified using FISH (Fig. 4A, B). Based on these results, we hypothesized that GAS6-AS1 functioned as a ceRNA. Thus, RNA immunoprecipitation (RIP) assays were conducted using an anti-Ago2 antibody. The results demonstrated that endogenous GAS6-AS1 was preferentially enriched in Ago2-RIPs compared to control IgG-RIPs (Fig. 4C). These findings suggested that GAS6-AS1 may serve as a ceRNA that promoted CRC development and progression.

GAS6-AS1 targeted miRNAs were predicted using the starbase database and we obtained 15 miRNAs that may bind with GAS6-AS1. We made use of TCGA database to analyze the expression difference and prognosis of the 15 miRNAs (Additional file 3: Fig. S1A). The results showed that five of them were highly expressed in normal tissues compared with colon cancer tissues, including miR-370-3p, miR-3173-5p, miR-1296-5p, miR-324-3p and miR-491-5p. Further survival analysis suggested that miR-370-3p and miR-1296-5p may play an anti-cancer role in colon cancer (Additional file 3: Fig. S1B). Therefore, we chose miR-370-3p and miR-1296-5p for further experiments. To verify whether GAS6-AS1 could bind with miR-370-3p and miR-1296-5p, Ago2-RIP assays and dual-luciferase reporter assays were performed. The Ago2-RIP assay demonstrated GAS6-AS1 higher enrichment in the miR-370-3p or miR-1296-5p mimic groups than in the NC mimic group (Fig. 4D, E). Dual-luciferase reporter assays demonstrated that upregulating miR-370-3p/miR-1296-5p suppressed the luciferase activity of the wild-type GAS6-AS1 reporter plasmid, but not the GAS6-AS1-370Mut or GAS6-AS1-1296Mut vectors (Fig. 4F, G). qRT-PCR also showed that the miR-370-3p and miR-1296-5p levels in CRC cells were negatively regulated by GAS6-AS1 (Fig. 4H, I). These findings confirmed that GAS6-AS1 directly bind to miR-370-3p and miR-1296-5p.

**Fig. 5:**
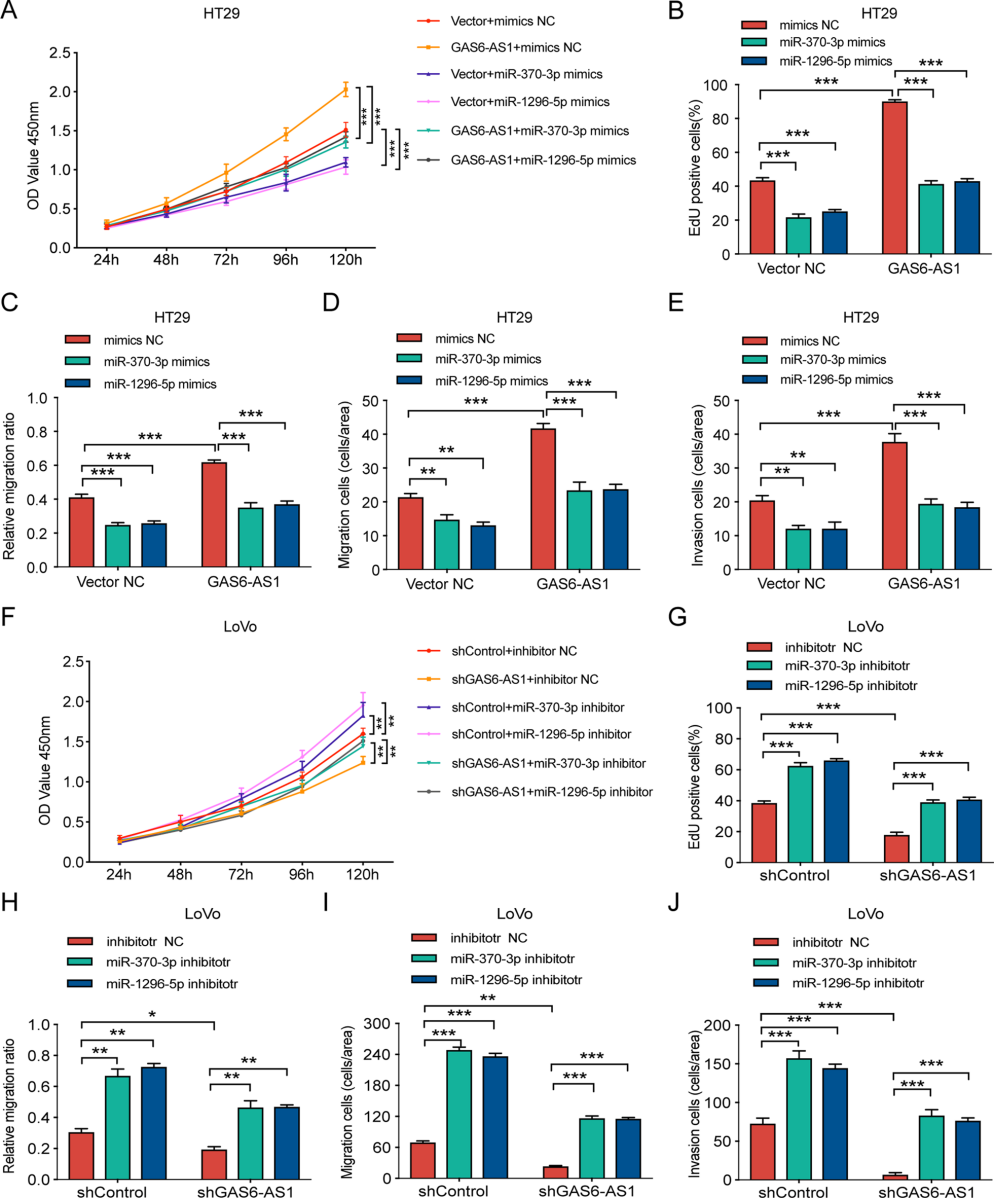
The function of GAS6-AS1 is mediated by miR-370-3p and miR-1296-5p. **A**–**E** miR-370-3p and miR-1296-5p mimics suppressed the proliferation, migration and invasion of HT29 cells, whereas miR-370-3p and miR-1296-5p mimics partially offset the promotion effects of GAS6-AS1 on HT29 cells’ proliferation, migration and invasion. **F**–**J** miR-370-3p and miR-1296-5p inhibitors promoted the proliferation, migration and invasion of LoVo cells, whereas miR-370-3p and miR-1296-5p inhibitors partially reversed the inhibitory effects of shGAS6-AS1 on LoVo cells’ proliferation, migration and invasion. ** *P* < 0.01, *** *P* < 0.001

To verify whether GAS6-AS1 regulated CRC cell viability and mobility via miR-370-3p and miR-1296-5p, we performed rescue experiments. In vitro functional experiments demonstrated that CRC cell growth, migration, and invasion decreased after miR-370-3p/miR-1296-5p upregulation. In addition, miR-370-3p/miR-1296-5p partially reversed GAS6-AS1-mediated cell proliferation, migration, and invasion (Fig. 5A–E, Additional file 4: Fig. S2A, C). Moreover, inhibiting miR-370-3p or miR-1296-5p promoted CRC cell proliferation, migration, and invasion, whereas downregulating miR-370-3p or miR-1296-5p reversed the effect of GAS6-AS1 knockdown (Fig. 5F–J, Additional file 4: Fig. S2D, F). These results suggested that GAS6-AS1 promoted the CRC oncogenesis via sponging miR-370-3p/miR-1296-5p.

### GAS6-AS1 decoys miR-370-3p/miR-1296-5p to regulate TRIM14

**Fig. 6:**
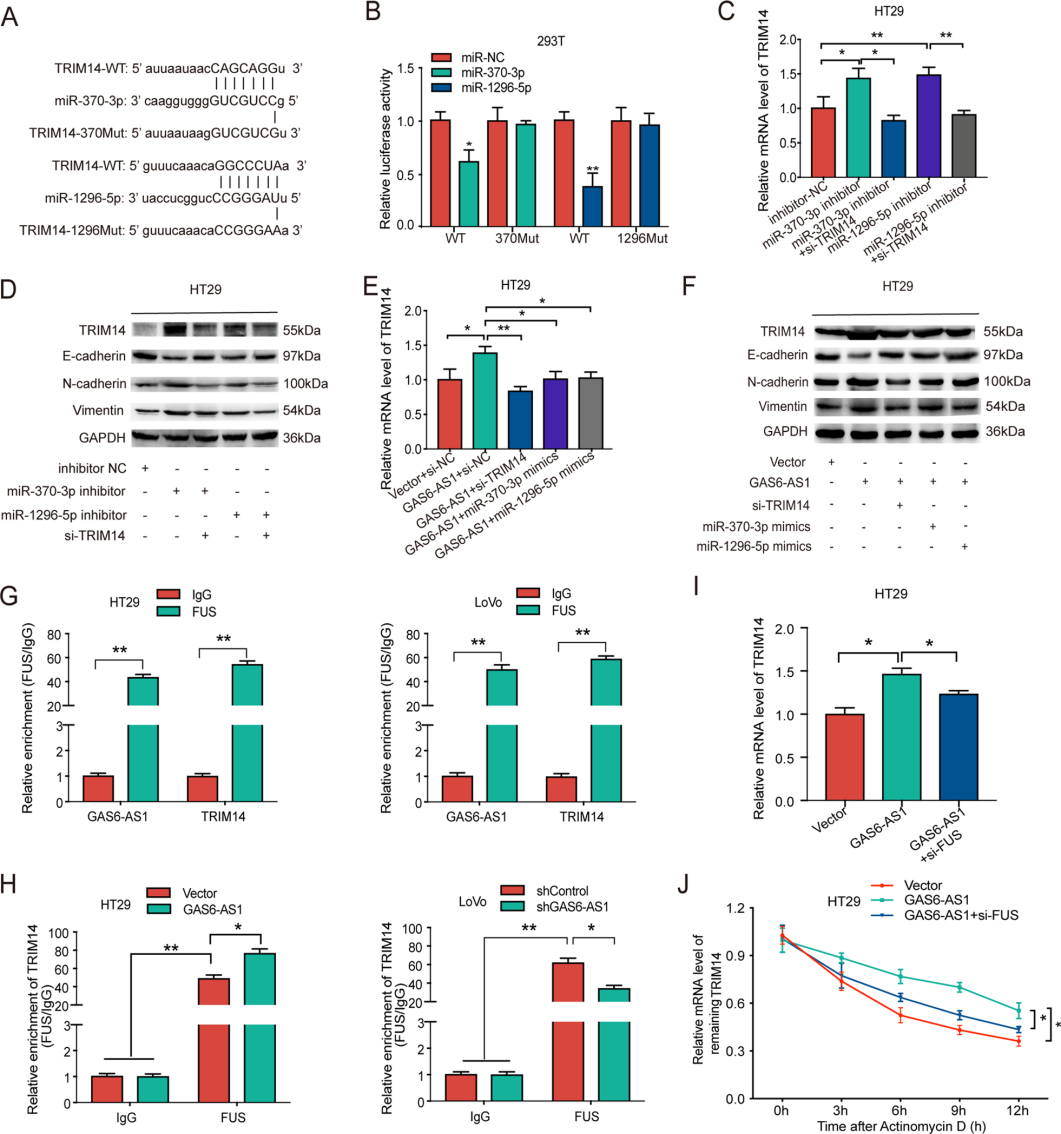
GAS6-AS1 regulats TRIM14 via ceRNA network and FUS-dependent manner. **A**, **B** miR-370-3p/miR-1296-5p and TRIM14 binding sequences and TRIM14 mutation sequences. The luciferase activities in 293T cells co-transfected with wild-type (WT) or mutant (370Mut/1290Mut) TRIM14 plasmid together with miR-370-3p or miR-1296 mimic or miR-NC. **C** The mRNA level of TRIM14 in indicated CRC cells were evaluated by qRT-PCR. **D** The protein level of TRIM14, E-cadherin, N-cadherin and Vimentin in indicated CRC cells were evaluated by western blot. **E** The mRNA level of TRIM14 in indicated CRC cells were evaluated by qRT-PCR. **F** The protein level of TRIM14, E-cadherin, N-cadherin and Vimentin in indicated CRC cells were evaluated by western blot. **G** RIP assay verified the interaction of GAS6-AS1 and TRIM14 mRNA with FUS. **H** RIP assay evaluated the impact of GAS6-AS1 on FUS-interacted TRIM14 mRNA. **I** qRT-PCR detected the expression of TRIM14 in indicated CRC cells. **J** TRIM14 expression after actinomycin D treatment for indicated times was tested by qRT-PCR. * *P* < 0.05, ** *P* < 0.01

To identify the genes that shared complementary binding sites of miR-370-3p and miR-1296-5p with GAS6-AS1, TargetScan was used to predict the common target genes of miR-370-3p and miR-1296-5p. Combined with a literature search, the TargetScan results predicted the common target gene, TRIM14. Previous reports revealed that higher TRIM14 levels in CRC contribute to disease progression [21, 22]. Therefore, TRIM14 was chosen for further analysis. Luciferase reporter assays indicated that miR-370-3p or miR-1296-5p upregulation reduced the luciferase activity of the wild-type TRIM14 reporter plasmid, but not of the TRIM14-370Mut or TRIM14-1296Mut vectors (Fig. 6A, B).

Moreover, TRIM14 mRNA levels in CRC cells were elevated after miR-370-3p or miR-1296-5p knockdown (Fig. 6C). Western blot analysis confirmed higher TRIM14 expression after miR-370-3p or miR-1296-5p knockdown (Fig. 6D), suggesting that miR-370-3p/miR-1296-5p negatively regulate TRIM14 levels in CRC cells. We also assessed the expression of E-cadherin, N-cadherin, and vimentin, which are EMT-associated markers. Notably, downregulating miR-370-3p or miR-1296-5p caused significant de-repression of E-cadherin and increased N-cadherin and vimentin levels. In addition, TRIM14 knockdown rescued the effects of miR-370-3p/miR-1296-5p inhibition in CRC cells (Fig. 6D). Thus, miR-370-3p/miR-1296-5p directly targeted TRIM14 to inhibit EMT in CRC cells.

We also observed high TRIM14 levels in CRC cells upon GAS6-AS1 overexpression, whereas TRIM14 knockdown or miR-370-3p/miR-1296-5p upregulation partially reversed the effect of GAS6-AS1 in CRC cells. In addition, GAS6-AS1 overexpression significantly decreased E-cadherin levels and increased N-cadherin and vimentin expression. Likewise, knocking down TRIM14 or upregulating miR-370-3p/miR-1296-5p reversed the changes caused by GAS6-AS1 (Fig. 6E, F). Thus, the results indicated that GAS6-AS1 could adsorb miR-370-3p/miR-1296-5p to upregulate TRIM14 and promote EMT in CRC cells.

### GAS6-AS1 stabilizes TRIM14 mRNA by interacting with FUS

Emerging evidence shows that RNA-binding proteins (RBPs), including FUS, maintain mRNA stability by interacting with lncRNAs [23]. Using the Starbase database, we predicted that GAS6-AS1 and TRIM14 both bind to FUS. RIP assays were performed to confirm this prediction. Both GAS6-AS1 and TRIM14 were harvested using FUS co-immunoprecipitation (Fig. 6G). Moreover, the level of TRIM14 mRNA bound to FUS significantly increased when GAS6-AS1 was upregulated but decreased when GAS6-AS1 knockdown (Fig. 6H). Meanwhile, qRT-PCR showed that overexpressing GAS6-AS1 increased the mRNA level of TRIM14, which was partially offset by downregulating FUS (Fig. 6I). In addition, GAS6-AS1 upregulation delayed TRIM14 mRNA degradation, whereas FUS knockdown partially rescued this effect (Fig. 6J). Based on these findings, GAS6-AS1 maintained TRIM14 mRNA stability by recruiting FUS.

### TRIM14 contributes to GAS6-AS1-mediated cell proliferation, migration, and invasion

**Fig. 7:**
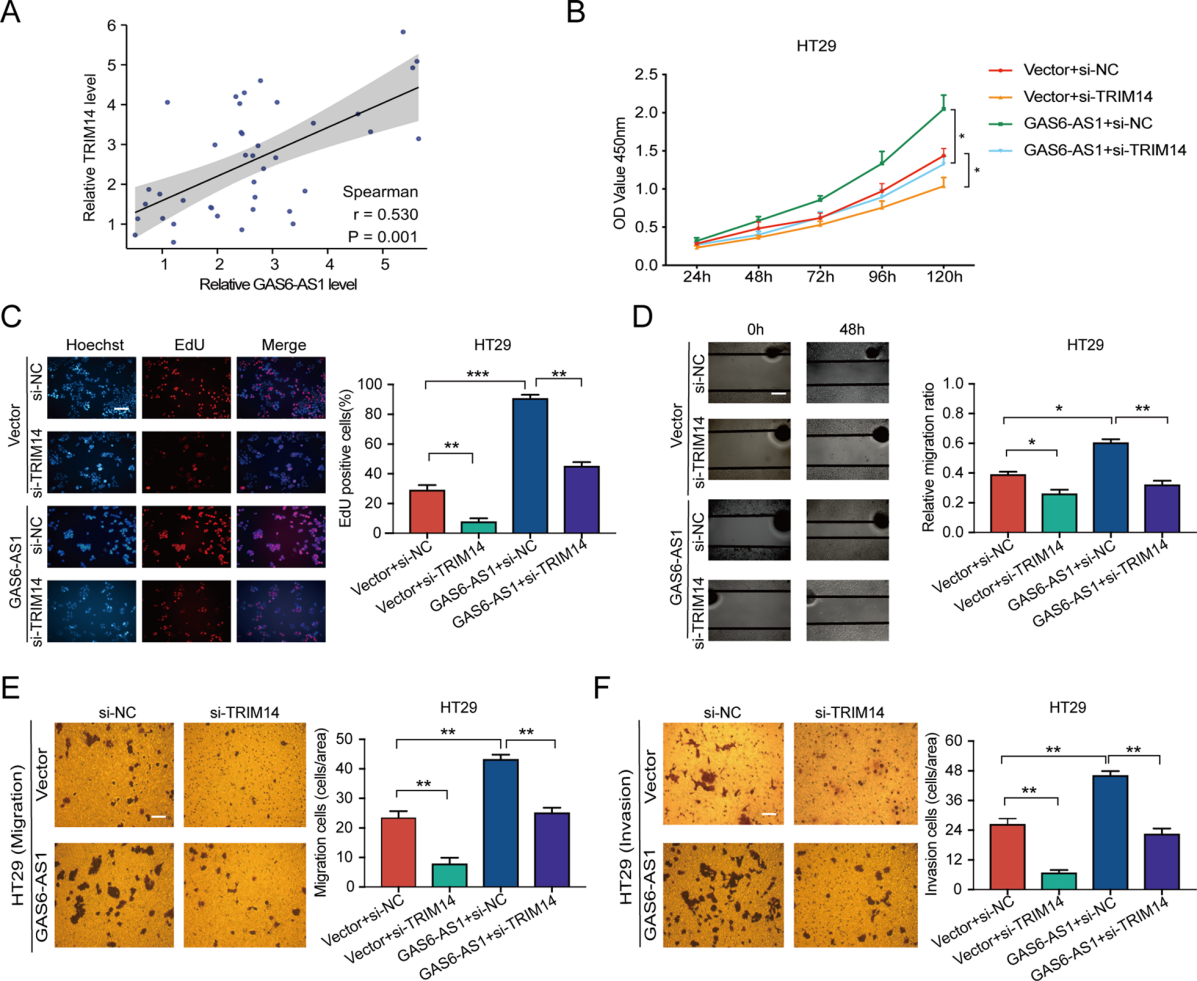
GAS6-AS1 promotes CRC development via regulating TRIM14**. A** The correlation between GAS6-AS1 and TRIM14 was analyzed by Spearman’s correlation analysis. **B**, **C** CCK8 and EdU assays revealed that GAS6-AS1 overexpressing-caused proliferation promoting could be reversed by silencing TRIM14. **D**–**F** Scratch and transwell assays revealed that the migration and invasion effects of GAS6-AS1 on CRC cells could be reversed by silencing TRIM14. (scale bar: 200 μm for EdU assay, 50 μm for wound healing assay, 100 μm for Transwell assay). * *P* < 0.05, ** *P* < 0.01, *** *P* < 0.001

To evaluate the correlation between GAS6-AS1 and TRIM14 expression in CRC tissues, TRIM14 levels in 40 CRC cases were analyzed by qRT-PCR for correlation analysis. The results showed a positive correlation between GAS6-AS1 and TRIM14 (Fig. 7A). Further rescue experiments were performed to verify whether GAS6-AS1 promoted tumor progression in a TRIM14-dependent manner. CCK8 and EdU assays revealed that TRIM14 downregulation partially reversed the effects of GAS6-AS1 overexpression on CRC cell proliferation (Fig. 7B, C). Scratch, transwell migration, and transwell invasion experiments demonstrated that TRIM14 knockdown rescued the effects of GAS6-AS1 overexpression on CRC cell migration and invasion (Fig. 7D–F). These findings demonstrated that GAS6-AS1 promoted CRC progression via TRIM14.

## Discussion

Compelling evidence indicated that lncRNAs play significant roles in CRC pathogenesis and have great value for CRC diagnosis and treatment [24, 25]. These abnormal lncRNAs contributed to various behaviors in CRC cells, including proliferation, apoptosis, metastasis, drug resistance, etc. Despite the unclear function of lncRNA GAS6-AS1 in tumors, reports showed that it was significantly elevated in gastric cancer tissues and drived progression of gastric cancer by activating GAS6 [9]. GAS6-AS1 levels were elevated in hepatocellular carcinoma, thereby promoting disease progression [10]. Moreover, GAS6-AS1 facilitated breast cancer malignancy via the PI3K/AKT pathway [11]. GAS6-AS1 is overexpressed in acute myeloid leukemia (AML) and promotes the progression of AML through the YBX1/MYC axis [15]. Besides, GAS6-AS1 linhibited the progression of lung adenocarcinoma and low GAS6-AS1 levels are associated with poor prognosis of patients [12–14]. However, the role of GAS6-AS1 in CRC has not been explored. This prompted us to examine the effects and potential mechanisms of GAS6-AS1 in CRC.

We first mined and analyzed the TCGA-COAD dataset in the TCGA database, which showed that GAS6-AS1 levels were higher in colon tissues than in normal. GAS6-AS1 expression was positively correlated with unfavorable clinicopathological factors in colon cancer. Further, patients with higher GAS6-AS1 levels had worse prognosis. By examining the GAS6-AS1 levels in 40 pairs of CRC tissues, we verified that the GAS6-AS1 level in CRC tissues was higher than that in adjacent tissues. Functional experiments demonstrated that GAS6-AS1 promoted CRC growth and metastasis in vitro and in vivo. These findings implicated GAS6-AS1 as a pro-tumorigenic lncRNA which involved in tumorigenesis and CRC progression.

ceRNA is an important mechanism by which lncRNAs participate in various cellular processes. Different RNAs sharing the same miRNA response element sequence competitively bind to the same miRNA, thereby forming a complex RNA regulatory network that regulates respective miRNA expression and co-interactions, ultimately influencing biological processes [26–29]. Numerous lncRNAs are associated with tumorigenesis and CRC development through ceRNA mechanisms. For instance, the lncRNA CACS19 promotes CRC progression by binding to miR-140-5p, which consequently upregulates CEMIP [30]. LncRNA PVT1 promotes CRC progression by sponging miR-30d-5p/miR-45 to regulate RUNX2 [31, 32]. Moreover, lncRNA CACS15 promotes oxaliplatin resistance in CRC cells by competitively binding to miR-145 and effectively upregulating ABCC1 expression [33]. The lncRNA TUG1 regulates the resistance of CRC cells to 5-FU by sponging miR-197-3p to upregulate TYMS [34]. Here, we show that GAS6-AS1 is located in the cytoplasm and nuclei of CRC cells and potentially exerts its function by regulating TRIM14, since it acts as a ceRNA to sponge miR-370-3p/miR-1296-5p.

Although the roles of miR-370-3p and miR-1296-5p in CRC remain unclear, miR-370-3p was found to suppress glioma cell proliferation and induce cell cycle arrest [35]. Additionally, miR-370-3p impeded bladder cancer cell invasion by suppressing Wnt7a expression, thus inhibited classical Wnt/β-catenin signal transduction and matrix metalloproteinase 10 (MMP10) levels [36]. MiR-1296-5p has been found to inhibit gastric cancer progression by suppressing CDK6 and EGFR [37]. Elsewhere, miR-1296-5p inhibited the viability of ERBB2-positive breast cancer cells by targeting the ERB2/mTORC1 pathway [38] and inhibits osteosarcoma development by targeting Notch [39]. These findings demonstrated that miR-370-3p and miR-1296-5p may act as antitumor molecules in cancers. Indeed, our results demonstrated that miR-370-3p and miR-1296-5p played a tumor-suppressive role in CRC. We confirmed that the interaction between GAS6-AS1 and miR-370-3p/miR-1296-5p and the pro-tumorigenic effect of GAS6-AS1 in CRC can be partially reversed by overexpressing miR-370-3p/miR-1296-5p.

TRIM14 was predicted as a potential target for miR-370-3p and miR-1296-5p, which was confirmed by luciferase reporter assays. Furthermore, upregulating miR-370-3p or miR-1296-5p decreased TRIM14 levels in CRC cells. Notably, TRIM14 belongs to the tripartite motif (TRIM) family and contributes to various biological processes. A previous report revealed that TRIM14 was highly expressed in a human immunodeficiency virus-related non-Hodgkin's lymphoma [40]. Additional studies revealed that TRIM14, which was a mitochondrial adapter that promotes innate immune signal transmission, participated in host defenses against viral infection [41–43]. TRIM14 was upregulated during inflammatory stimulation with TNF-α, IL-1β, and LPS, and its overexpression promoted monocyte and endothelial cell adhesion [43]. Another study found that TRIM14 was a novel regulator of the non-specific NF-κB signaling pathway [44]. Moreover, TRIM14 expression were found upregulated in gastric cancer [45]. oral squamous cell carcinoma [46], tongue cancer [47], breast cancer [48], hepatocellular carcinoma [49], osteosarcoma [50] and glioma [51], and function as an oncogene in these cancers. Furthermore, TRIM14 was elevated in CRC tissues and promoted the migration and invasion of CRC cells via the SPHK1/STAT3 pathway [21]. High TRIM14 expression was closely correlated with poor prognosis of CRC patients, increased cell growth, and inhibited CRC cell apoptosis via the PTEN/Akt pathway [22]. In this study, we confirmed that GAS6-AS1 could promote CRC progression via GAS6-AS1-miR-370-3p/miR-1296-TRIM14 axis.

An increasing number of studies show that RBPs are involved in the regulation of gene expression by lncRNAs, binding to RBP is one of the main mechanisms by which lncRNAs play a role in the pathological process of cancers [52]. Here, in addition to the mechanism of acting through ceRNA network, we confirmed that GAS6-AS1 stabilized TRIM14 mRNA through FUS-mediated way, thus further improving the molecular mechanism of GAS6-AS1 facilitating CRC development via mediating TRIM14.

To our knowledge, the present study is the first to report the clinical significance and function of GAS6-AS1 in CRC. Furthermore, functional and mechanistic experiments demonstrated the potential role of GAS6-AS1 as a ceRNA, which involved in accelerating CRC growth and metastasis via sponging miR-370-3p/miR-1296-5p to regulate TRIM14. On the other hand, GAS6-AS1 stabilizes TRIM14 mRNA by recruiting FUS. Therefore, GAS6-AS1 may be a potential biomarker and therapeutic target for CRC treatment. However, our study has some limitations, including analysis using a single dataset and a limited number of collected tissue samples. In future, we will explore the biological functions of GAS6-AS1 in CRC in more detail.

## Supplementary Information

**Additional file 1: Table S1.** Primers.

**Additional file 2: Table S2.** Characteristics of TCGA-COAD patients.

**Additional file 3: Figure S1.** The expression difference and prognosis of miRNAs that may bind to GAS6-AS1 from the TCGA database. **A** The expression difference of 15 miRNAs in TCGA-COAD dataset. **B** Kaplan–Meier analysis of overall survival of the miRNAs. ns: none significance, * *P* < 0.05, ** *P* < 0.01, **** *P* < 0.0001.

**Additional file 4: Figure S2.** The Representative images of the EdU, Scratch healing, and Transwell assays matched to Fig. 6. (scale bar: 200 μm for EdU assay, 50 μm for wound healing assay, 100 μm for Transwell assay).

## Data Availability

The data and materials in this study are available from the corresponding author on request.
