# Supplementary material for: LncRNA GAS6-AS1 facilitates tumorigenesis and metastasis of colorectal cancer by regulating TRIM14 through miR-370-3p/miR-1296-5p and FUS
Source: J Transl Med. 2022 Aug 12;20:356. doi: 10.1186/s12967-022-03550-0 (PMC9373365; doi:10.1186/s12967-022-03550-0)
Supplement: Supplementary file 1 — Additional file 1: Table S1. Primers. [file 12967_2022_3550_MOESM1_ESM.pdf]

Table S1. The primer sequences used in this study

Primer	Sequence
LncRNA GAS6-AS1-F1	5’- ACCATGGAGGGATGTCTGGG-3’
LncRNA GAS6-AS1-R1	5’- CCCAGCCCTTTGCATCACAT-3’
TRIM14-F	5’- GCTTCTTTAAGGGCCTCGT-3’
TRIM14-R	5’- GTCTGAGAGCTGGCAGTTTAT-3’
β-actin-F	5’-CTCACCATGGATGATGATATCGC-3’
β-actin-R	5’-AGGAATCCTTCTGACCCA TGC-3’
U6-F	5’-CTCGCTTCGGCAGCACATA-3’
U6-R	5’-AACGATTCACGAATTTGCGT-3’
miR-370-3p	5’- GCCTGCTGGGGTGGAAC-3’
miR-1296-5p	5’- TTAGGGCCCTGGCTCCATC-3’
U6	5’- GAAGGATGACACGCAAATTCG-3’
