# Supplementary material for: LncRNA GAS6-AS1 facilitates tumorigenesis and metastasis of colorectal cancer by regulating TRIM14 through miR-370-3p/miR-1296-5p and FUS
Source: J Transl Med. 2022 Aug 12;20:356. doi: 10.1186/s12967-022-03550-0 (PMC9373365; doi:10.1186/s12967-022-03550-0)
Supplement: Supplementary file 2 — Additional file 2: Table S2. Characteristics of TCGA-COAD patients. [file 12967_2022_3550_MOESM2_ESM.pdf]

**Table S2. Characteristics of TCGA-COAD patients**

| Clinical characteristics | | Total (446) | % |
|---|---|---|---|
| Gender | | | |
| | Female | 211 | 47.3 |
| | Male | 235 | 52.7 |
| Age | | | |
| | <60 | 121 | 27.1 |
| | ≥60 | 325 | 72.9 |
| T | | | |
| | T1 | 9 | 2.0 |
| | T2 | 76 | 17.0 |
| | T3 | 304 | 68.2 |
| | T4 | 57 | 12.8 |
| N | | | |
| | N0 | 265 | 59.4 |
| | N1 | 102 | 22.9 |
| | N2 | 79 | 17.7 |
| M | | | |
| | M0 | 329 | 84.4 |
| | M1 | 61 | 15.6 |
| Stage | | | |
| | Ⅰ | 74 | 16.9 |
| | Ⅱ | 178 | 40.5 |
| | Ⅲ | 126 | 28.7 |
| | Ⅳ | 61 | 13.9 |
| MSI Status | | | |
| | MSH | 83 | 18.6 |
| | MSL/MSS | 363 | 81.4 |
