# Supplementary material for: LncRNA GAS6-AS1 facilitates tumorigenesis and metastasis of colorectal cancer by regulating TRIM14 through miR-370-3p/miR-1296-5p and FUS
Source: J Transl Med. 2022 Aug 12;20:356. doi: 10.1186/s12967-022-03550-0 (PMC9373365; doi:10.1186/s12967-022-03550-0)
Supplement: Supplementary file 3 — Additional file 3: Figure S1. The expression difference and prognosis of miRNAs that may bind to GAS6-AS1 from the TCGA database. A The expression difference of 15 miRNAs in TCGA-COAD dataset. B Kaplan–Meier analysis of overall survival of the miRNAs. ns: none significance, * P < 0.05, ** P < 0.01, **** P < 0.0001. [file 12967_2022_3550_MOESM3_ESM.pdf]

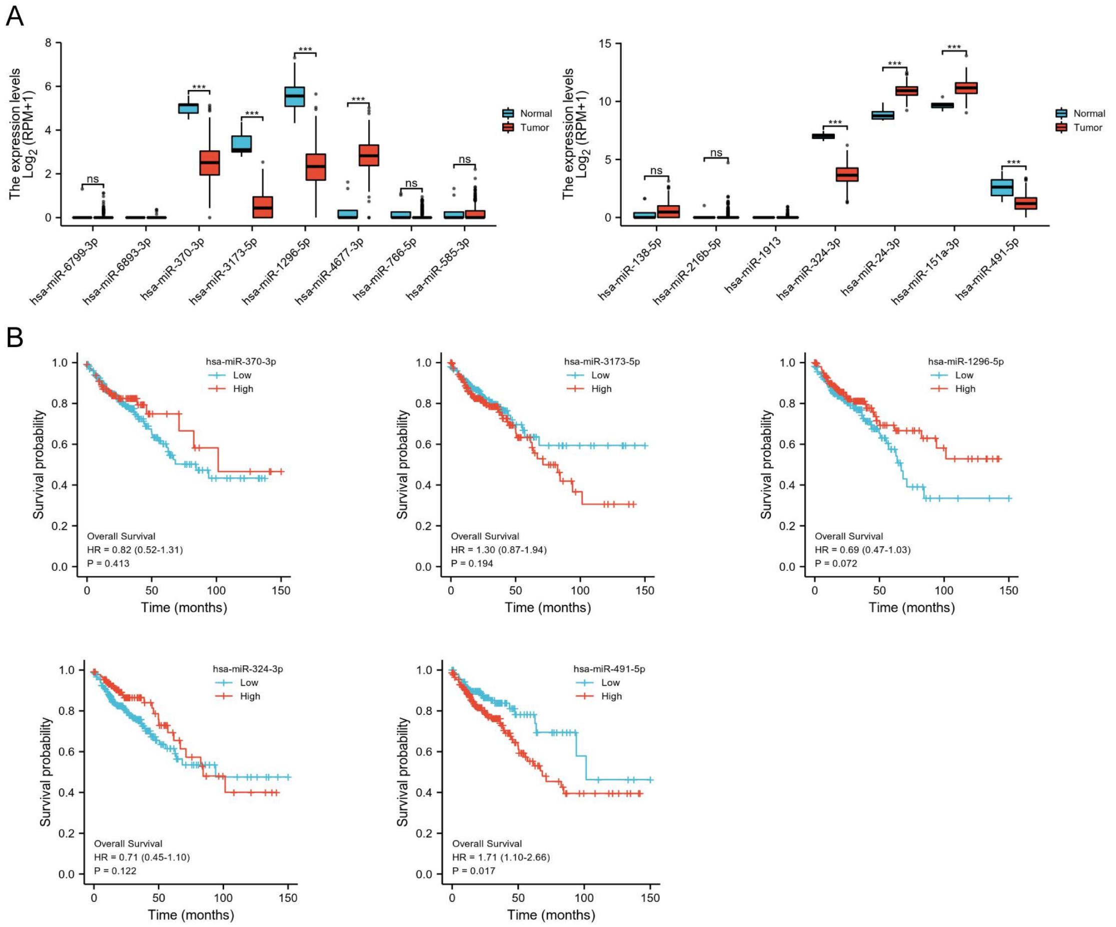

Figure S1. The expression difference and prognosis of miRNAs that may bind to GAS6-AS1 from the TCGA database

(A) The expression difference of 15 miRNAs in TCGA-COAD dataset. (B) Kaplan-Meier analysis of overall survival of the miRNAs. ns: none significance, * $P < 0.05$, ** $P < 0.01$, **** $P < 0.0001$.
