# Supplementary material for: LncRNA GAS6-AS1 facilitates tumorigenesis and metastasis of colorectal cancer by regulating TRIM14 through miR-370-3p/miR-1296-5p and FUS
Source: J Transl Med. 2022 Aug 12;20:356. doi: 10.1186/s12967-022-03550-0 (PMC9373365; doi:10.1186/s12967-022-03550-0)
Supplement: Supplementary file 4 — Additional file 4: Figure S2. The Representative images of the EdU, Scratch healing, and Transwell assays matched to Fig. 6. (scale bar: 200 μm for EdU assay, 50 μm for wound healing assay, 100 μm for Transwell assay). [file 12967_2022_3550_MOESM4_ESM.pdf]

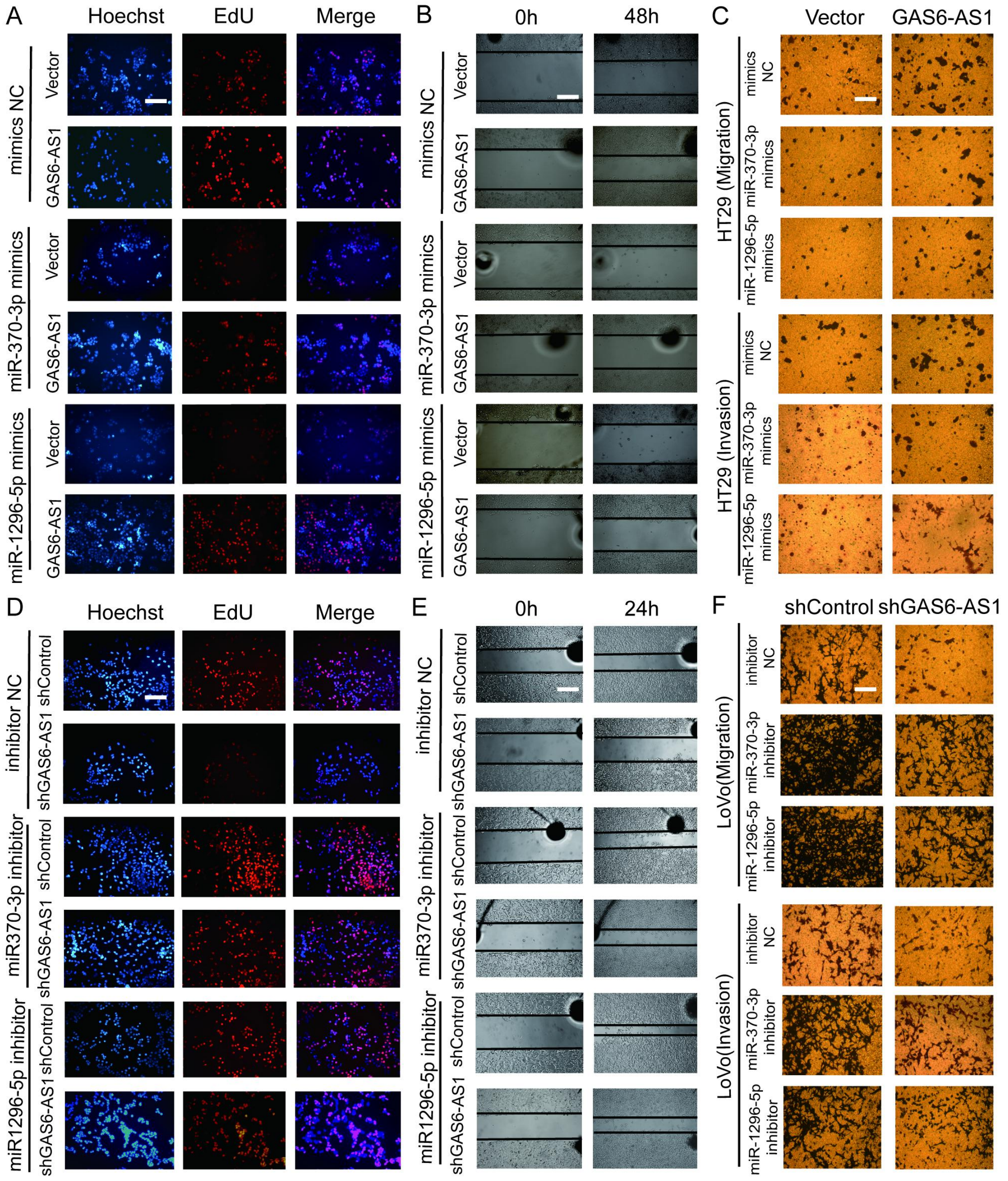


**Figure S2. The representative images of the EdU, scratch healing, and transwell assays matched to Fig 5**

(scale bar: 200 μm for EdU assay, 50 μm for wound healing assay, 100 μm for transwell assay).
